# On Trapped Flux in a Small Crystal of CaKFe$_4$As$_4$ and Implications for High-Pressure Hydrides

**DOI:** 10.1007/s10948-025-06948-1

**Published:** 2025-03-25

**Authors:** J. E. Hirsch, F. Marsiglio

**Affiliations:** 1 Department of Physics, University of California, San Diego, La Jolla, CA 92093-0319 USA; 2 Department of Physics, University of Alberta, Edmonton, Alberta T6G 2E1 Canada

**Keywords:** Hydride superconductors, Trapped flux, Zero field cooling, Quadratic versus linear behavior, Tiny samples

## Abstract

In recent work (Bud’ko et al. Supercond. Sci. Technol. **37**, 065010 2024), Bud’ko et al. present experimental results for trapped magnetic flux for a tiny sample of a type II superconductor, $CaKFe_4As_4$. The paper aims to provide evidence in support of the interpretation that similar measurements performed in samples of hydrogen-rich materials under high pressure by Minkov et al. (Nat. Phys. **19**, 1293 2023) are conclusive evidence (Eremets Nat. Sci. Rev. **11**, nwae047 2024) for superconductivity in hydrides under pressure. Here, we point out that the new evidence presented by Bud’ko et al. (Supercond. Sci. Technol. **37**, 065010 2024) further supports our interpretation (Hirsch and Marsiglio J. Supercond. Nov. Magn. **35**, 3141–3145 2022; Hirsch and Marsiglio Phys. C **620**, 1354500 2024) that the reported measurements of trapped flux on hydrides under pressure (Minkov et al. Nat. Phys. **19**, 1293 2023) are not consistent with what would be expected from a superconducting sample.

## introduction

**Fig. 1:**
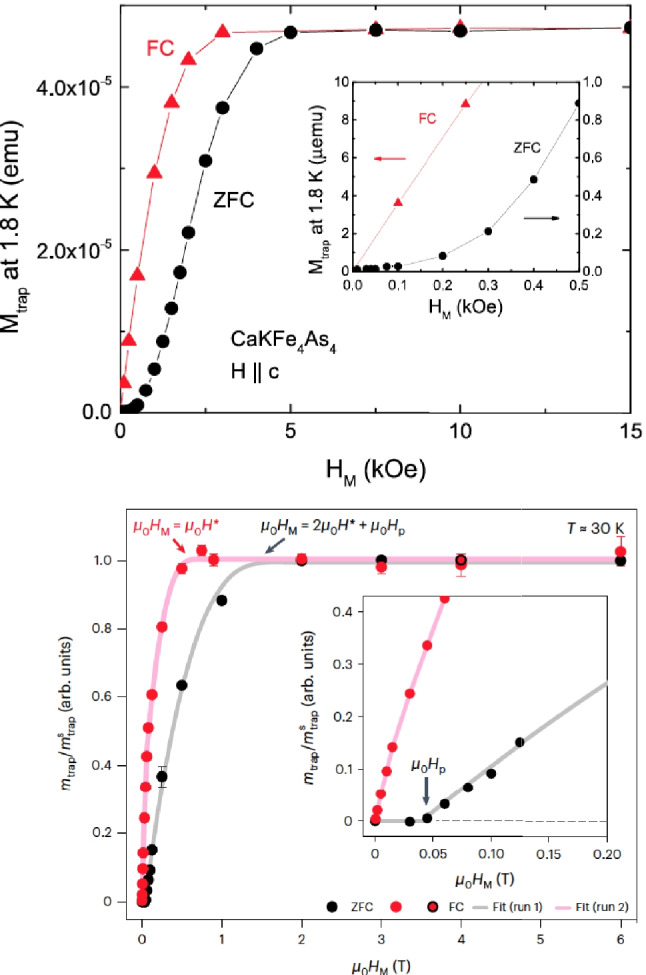
Top panel: trapped magnetic moment for a sample of superconducting CaKFe$_4$As$_4$ after applied field is removed in zero field cooling (ZFC, black points) and field cooling (FC, red points) protocols, from Ref. [1]. Bottom panel: same for a sample of H$_3$S under high pressure, from Ref. [2]. In both panels, lines were drawn through the points to guide the eye by the authors of Refs. [1, 2]

In the paper Supercond. Sci. Technol. 37 (2024) 065010 [1], Bud’ko et al. present results of flux trapping measurements for a tiny sample of superconducting CaKFe$_4$As$_4$. The aim of the work is to provide support for the interpretation that similar measurements on hydrides under high pressure [2] are evidence that hydrides under high pressure are superconductors. Figure 1 shows measurements of a tiny sample of the superconducting material on the top panel [1] and on the bottom panel of a sample of similar size of H$_3$S [2]. Superficially, the two panels may look qualitatively similar. In this paper, we point out that, in fact, they are qualitatively different. But first, some historical context.

Minkov, Ksenofontov, Bud’ko et al. have published results of experiments on hydrides under high pressure [2], which showed that when a magnetic field is applied to the material and subsequently removed, a remnant magnetization is detected, which they interpret as originating in “trapped flux.” They inferred from their measurements that the remnant magnetization is produced by electric currents flowing in the material that do not decay with time, providing “conclusive evidence” [3] that the material is a superconductor.

In Ref. [4], we argued that the measurements of Minkov et al. [2] do not support superconductivity because the observed dependence of trapped moment under zero field cooling (ZFC) versus magnetic field was linear rather than quadratic. We showed results of our calculations with a simple model showing that the expected behavior due to superconducting currents should be quadratic [4].

Subsequently, Bud’ko, Xu, and Canfield [6] did similar measurements as Minkov et al. [2] on known superconducting materials, using samples that were about 1000 times larger in volume than the samples used by Minkov et al. [6]. Bud’ko, Xu, and Canfield concluded [6] that their measurements supported the interpretation of the experiments by Minkov et al. [2].

However, we showed in Ref. [5] that the reported results by Bud’ko, Xu, and Canfield [6] strongly supported our original analysis and interpretation [4] of the Minkov et al. results on hydrides [2].

## Analysis

Our analysis of flux trapping experiments in Refs. [4, 5] was based on the Bean model, under the simplifying assumption that the critical current is uniform over the superconducting region. We recognize that this is a simplifying assumption and that real superconducting samples could show some variation of critical current as function of field magnitude and position in the sample, so we do not expect exact agreement of calculated results with measured results. Still, we expect that our model will capture the essential physics and yield approximately correct results for measurements of trapped flux in superconducting samples. In particular, an important aspect of the physics emphasized in Refs. [4, 5] is that under ZFC, the trapped magnetic moment predicted by our model and mandated on physical grounds should be quadratic and not linear as function of applied field.

The model, quantitatively described in Refs. [4, 5], has the following adjustable parameters: (i) $H^*$, the value of the magnetic field for which an applied magnetic field reaches the center of the sample, which is proportional to the critical current density; (ii) $H_p$, the threshold value of the magnetic field below which an applied field does not penetrate, which is the lower critical field corrected for demagnetization; and (iii) the value of the saturation trapped magnetic moment $m_s$.

In this paper, we show that, in fact, the most recent results of Bud’ko et al. [1] provide further confirmation of the validity of our analysis [4, 5] of the Minkov et al. experiments [2] and of our interpretation that the signals measured in hydrides are not due to superconductivity.

**Fig. 2:**
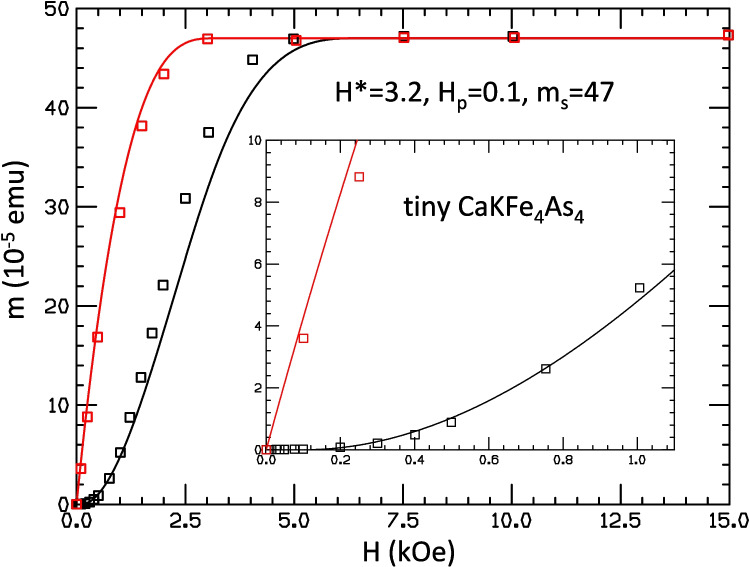
Theoretical fit to the experimental results of Ref. [1] for trapped flux in CaKFe$_4$As$_4$ (red and black lines for FC and ZFC, respectively) using the theoretical model of Refs. [4, 5]. Parameters used for the fit are given in the figure

**Fig. 3:**
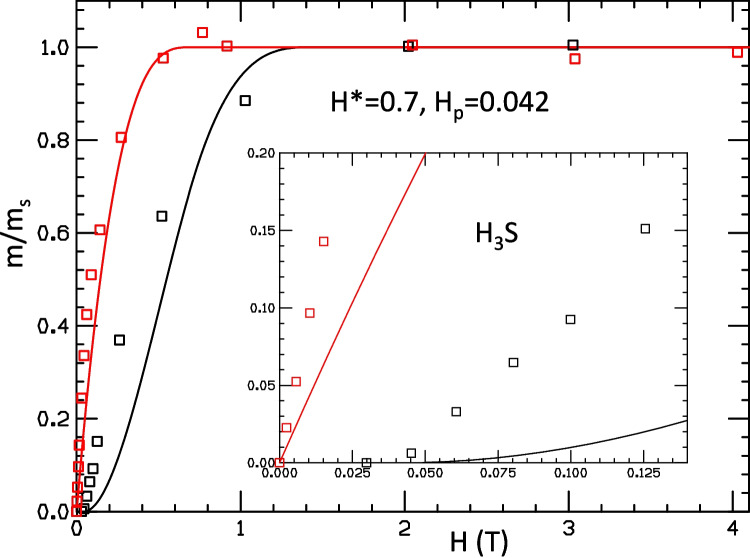
Theoretical fit to the experimental results of Ref. [2] for trapped flux in H$_3$S (red and black lines for FC and ZFC, respectively) using the theoretical model of Refs. [4, 5]. Parameters used for the fit are given in the figure. They were chosen to approximately match the field values where the magnetic moment reaches saturation

**Fig. 4:**
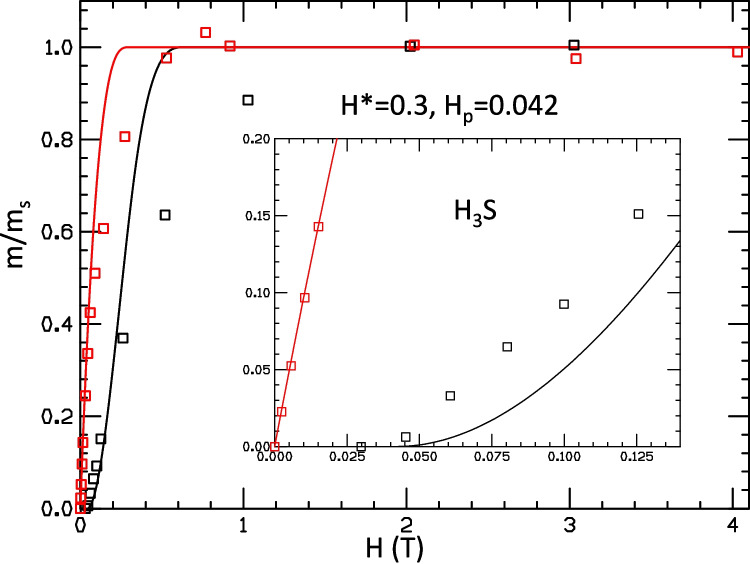
Theoretical fit to the experimental results of Ref. [2] for trapped flux in H$_3$S (red and black lines for FC and ZFC, respectively) using the theoretical model of Refs. [4, 5]. Parameters used for the fit are given in the figure. They were chosen to approximately match the low field behavior of the FC moment

**Fig. 5:**
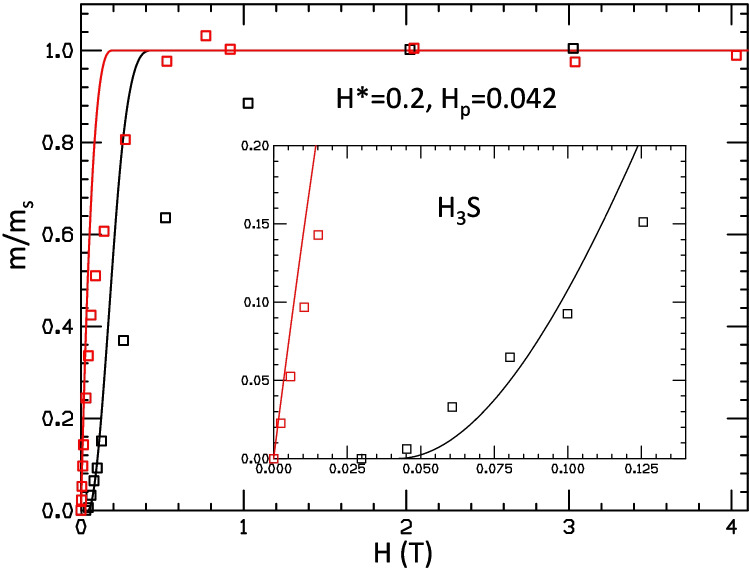
Theoretical fit to the experimental results of Ref. [2] for trapped flux in H$_3$S (red and black lines for FC and ZFC, respectively) using the theoretical model of Refs. [4, 5]. Parameters used for the fit are given in the figure. They were chosen to approximately match the low field behavior of the ZFC moment

Figure 2 shows comparison of the predictions of our model with the measured results on the superconducting sample studied in Ref. [1], with the values of the adjustable parameters shown in the figure. These values were chosen so as to give the best possible fit to the low field values of the ZFC trapped moment for low fields, which clearly shows supralinear behavior (quadratic) as seen in the inset of Fig. 2. It can be seen that these parameters also give a good fit to the FC moment at low field (inset of Fig. 2). Furthermore, they provide a reasonable fit to the behavior over the entire field range, as shown in the main body of Fig. 2. There are some deviations from the measured values at intermediate field values, but it can be seen that the values where the magnetic moment reaches saturation, which in our model is $H^*$ and $2H^*+H_p$ for FC and ZFC protocols, respectively, are not far from the values indicated by the experimental points.

In Figs. 3, 4, and 5, we attempt such fits for the hydride sample $H_3S$ using the measured values reported in Ref. [2]. The low field behavior of the ZFC moment gives a value of $H_p=0.042T$, as determined in Ref. [2], which we use in the three figures. We start in Fig. 3 by choosing $H^*$ to approximately fit the field values where the moment reaches saturation, which was determined to be $H^*=0.7T$ in Ref. [2]. The moment under ZFC reaches saturation at magnetic field approximately twice as large as the FC, as our model predicts. It can be seen in Fig. 3 that the red and black lines approximately fit the measured points near the values where the moments reach saturation. However, it can be seen in the inset of Fig. 3 that there is drastic disagreement with the behavior of the moments at small field, the theoretical curves giving values substantially lower than the measured ones, particularly for the ZFC protocol.

In Fig. 4, we pick $H^*$ so as to fit the low field behavior of the FC moment. This requires a much smaller value of $H^*$, namely $H^*=0.3T$. It can be seen that the low field values of the ZFC moment are still substantially underestimated. Furthermore, the overall behavior seen in the main body of the figure is in drastic disagreement with experiment, since the curves reach saturation substantially before the measured moments reach saturation, both for FC and ZFC protocols.

Finally, in Fig. 5, we pick an even smaller value of $H^*$, $H^*=0.2$, to attempt a better match to the low field ZFC moments. It can be seen that the ZFC moments are still not matched by the theory, because they do not follow the same functional form as the theory predicts, namely quadratic in field; nevertheless, we try to fit the behavior on average, with some points falling below, some above, the theoretical curve. The small field FC moment is now overestimated by the theoretical values, and as seen in the main panel of the figure, the overall disagreement with the measured values is stark both for FC and ZFC moments.

**Fig. 6:**
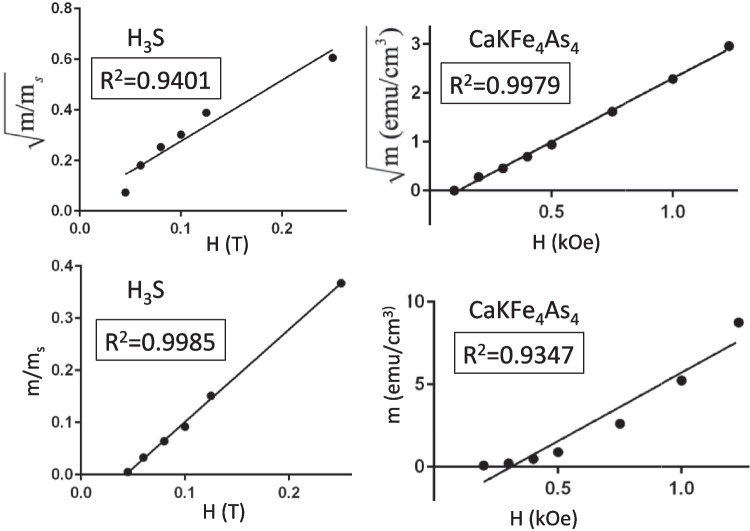
Top panels: Linear regression fits of square root of trapped moment versus magnetic field for H$_3$S [2] (top left panel) and for the tiny sample of CaKFe$_4$As$_4$ [1] (top right panel). It can be seen that for the superconductor (top right panel), the fit is very good, indicating quadratic behavior of trapped moment versus field as predicted by our model and by general physical arguments. Instead, for H$_3$S (top left panel), the fit is not good. Bottom panels: Linear regression fit of trapped moment versus magnetic field for H$_3$S (bottom left panel) and for CaKFe$_4$As$_4$ (bottom right panel). It can be seen that, for the superconductor (bottom right panel), the fit is not very good, indicating that the trapped moment under ZFC is not linear with magnetic field, as predicted by our model and general physical arguments. Instead, for H$_3$S (bottom left panel), the linear fit is excellent

As we also did in Ref. [5], in order to quantify the deviation of the low-field ZFC data for H$_3$S from the expected behavior of a superconductor, we show in Fig. 6 linear regression fits [7] to the square root and first power of the trapped moment versus magnetic field, respectively, for the two materials considered here. The coefficient $R^2$ measures how well the data fit the regression model, with $R^2=1$ being a perfect fit. It can be seen in the top panels that $R^2$ is larger than 0.99 for the superconductor, indicating an excellent fit to quadratic behavior of magnetic moment versus magnetic field, while for H$_3$S, the $R^2$ differs substantially from unity, indicating the observed behavior is not consistent with quadratic dependence of moment on field. Conversely, as seen in the bottom panels, a linear fit of magnetic moment vs field fits the H$_3$S data extremely well, with $R^2=0.9979$, and does substantially worse for the standard superconductor. This provides strong evidence that for the standard superconductor the behavior of magnetic moment versus field under ZFC is quadratic, just as we found in Ref. [5] for several other superconductors as well, while it is linear for H$_3$S.

## Effect of Pressure and Background Subtraction

**Fig. 7:**
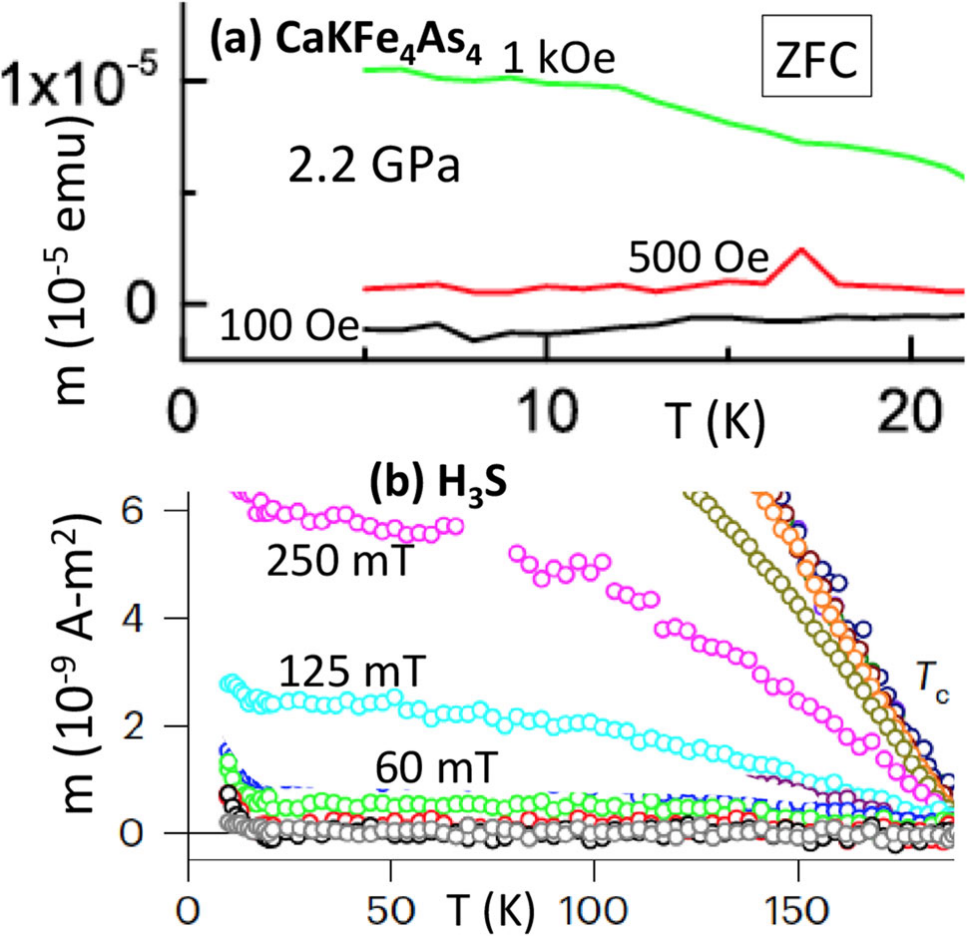
**a** From Fig. 5b of Ref. [2]: temperature-dependent trapped flux magnetization of CaKFe$_4$As$_4$ measured in H =0 using ZFC protocol in DAC under pressure of 2.2 GPa. The magnetic field was applied at base temperature $T=1.8K$, then removed. **b** From Fig. 1c of Ref. [2]: temperature-dependent trapped flux magnetization of H$_3$S measured in $\textrm{H} =0$ using ZFC protocol in DAC under pressure of 155 GPa. The magnetic field was applied at base temperature $T=10K$, then removed

For the sample under pressure, Ref. [1] does not report magnetic field dependence of the trapped moment with sufficient detail in their Fig. 6 to make it possible for us to fit the results as done in Fig. 2. Nevertheless, we can get some information from the temperature-dependent trapped moments reported in Fig. 5(b) of Ref. [1], shown here in Fig. 7a. It is very clear from Fig. 7a that the behavior of the ZFC moment with field in the DAC under pressure is very non-linear, just as it was for the sample at ambient pressure: in increasing the magnetic field from 500 Oe to 1 kOe, i.e., by 500 Oe, the increase in trapped moment seen in Fig. 7a is a factor of 5 times larger than in increasing the magnetic field from 100 to 500 Oe, i.e., by 400 Oe. This is in clear disagreement with what would be expected for a sample whose trapped moment behaved linearly like the hydride sample shown in the inset of Fig. 1 lower panel, which would show an increase in moment when going from 500 Oe to 1 kOe that is only a factor 1.25 larger than in going from 100 to 500 Oe. For comparison, Fig. 7b shows similar curves for H$_3$S from Fig. 1c of Ref. [2]. Here, the increase in the low temperature moment in going from 125 to 250 mT, i.e., an increase of 125 mT, is approximately twice the increase in going from 60 to 125 mT, i.e., an increase of 65 mT, as expected for linear behavior.

Furthermore, while the purpose of Ref. [1] is “*to address concerns about sample size and signal associated with trapped flux measurements in a DAC*,” i.e., under pressure, the authors of Ref. [1] have actually managed to raise concerns instead. They themselves have noted the large discrepancy in signal size in the normal state under 2.2 GPa pressure in the DAC (their Fig. 4a) compared to that at zero pressure without the DAC (their Fig. 1), and the accompanying increase in noise, possibly attributing these discrepancies to the presence of “*a fairly large background*” in the DAC (see the discussion on p. 4–5 of Ref. [1]). The authors include an Appendix B with figures produced from a commercial DAC—see the description of this commercial DAC plus accompanying software in Ref. [16] of Ref. [1]—but do not show background and sample + background results as a function of temperature or applied magnetic field. It would also have been useful to see more detailed results for the trapped field magnetization as a function of applied field in the DAC under pressure—only four unsaturated values are shown in their Fig. 6.

As one of the purposes of Ref. [1] is to convince the reader of the validity of hydride results presented in Ref. [2] (their Ref. [6]), it is unfortunate that there are “*important differences in protocols for trapped flux magnetization measurements between this work and* [6].” In particular, the presence of a “*remnant magnetic field...which depends on the superconducting magnet design and geometry as well as the history of magnetic field applied prior to the measurement*” is a disturbing source of uncertainty. One is left wondering about the possibility of spurious effects in both these measurements and those in the hydrides, Ref. [2], that could depend significantly on the background subtraction procedure that is applied, which is not clearly explained in Ref. [2].

Given that this group has considerable experience with CaKFe$_4$As$_4$ at even higher pressures (above 4 GPa) where this material is *not* superconducting [8], it would be of considerable interest to test for trapped flux under these higher pressures using the same background subtraction procedure as used for the lower pressures where it is superconducting. Another test to determine the role of the apparatus alone would be to redo the experiments at zero pressure *inside* the DAC, to better differentiate the roles of the actual pressure versus the presence of the DAC itself.

Ultimately, however, for a convincing demonstration of the utility of magnetization measurements on hydride materials in DACs, one would need to follow the same protocol in a known superconductor as was used in the hydrides and properly document the background subtractions that are required.

## Conclusion

In Ref. [1], the authors conducted trapped flux experiments on a sample of a known superconducting material, attempting to mimic the conditions in the experiments for hydrides under pressure [2], by picking a tiny sample of volume comparable to the hydride samples, and subjecting it to pressure in a diamond anvil cell.

Here, we have shown that our model [4] reproduces the observed behavior in this superconducting tiny sample remarkably well, as shown in Fig. 2. Upon picking the parameters in the model to fit the low field behavior of the ZFC moment, our model predicts the behavior of the small field FC moment as well as the overall behavior of the ZFC and FC moments including the values where the moments reach saturation remarkably well. The maximum discrepancy in the values of calculated moment vs measured moment in Fig. 2 is $25\%$. This indicates that the model captures the essential physics of trapped moments in type II superconductors even for tiny samples such as those used in the hydride experiments.

In contrast, we showed that our model cannot describe the behavior of the moments reported for the hydride sample H$_3$S [2] for any set of parameters in the model. Choosing $H^*=0.7T$ as was done in Ref. [2], to fit approximately the fields where the moments reach saturation, gives rise to major discrepancies in the calculated versus measured values for lower fields, as shown in Fig. 3; the discrepancy in the values of calculated moment vs measured moment in Fig. 3 is up to $800\%$, 30 times larger than for the superconducting sample in Fig. 2. If $H^*$ is instead chosen to approximately fit the low field behavior, there are major differences for the high field behavior, as shown in Figs. 4 and 5. Furthermore, the quadratic behavior of ZFC moment with field at small field that is seen in this tiny sample [1], as well as all the superconductor samples studied by these authors earlier [6], is perfectly fitted by our model. The linear behavior seen in the hydrides that is not fitted by our model is in stark contrast with the behavior of all the superconducting samples, including the sample studied in Ref. [1] in the DAC under pressure 2.2 GPa, as we discussed in Sect. 3.

Furthermore, Ref. [1] showed that when the tiny sample is measured in a DAC under pressure, significant other issues come into play. The magnetization under an applied field as well as the noise show large differences compared to the measurements without the DAC. To be relevant to the interpretation of hydride results, the protocols used, in particular background subtraction, should be the same. In particular, Ref. [1] emphasized the need for point-by-point background subtraction. It is not clear what was done in Ref. [2].

We conclude that the remnant moments measured in hydrides [2], shown here in Fig. 1 lower panel, do not show behavior that is consistent with the behavior seen in the tiny superconducting sample studied in Ref. [1], neither at ambient pressure nor under pressure. In our view, the most likely possibility is that the remnant moments measured in Ref. [2] originate in other phenomena or experimental artifacts unrelated to superconductivity, in accordance with the conclusions reached in our earlier papers on this subject [4, 5]. On the other hand, the possibility that hydrides under pressure are superconductors that trap magnetic flux in a non-standard way [9] cannot be ruled out. To support such a hypothesis, it would be useful to find other materials known to be superconducting that show linear behavior of trapped moment versus field under ZFC conditions as $H_3S$ does. It would also be useful to repeat the results for $H_3S$ shown in Fig. 1 bottom panel for other hydride samples, as well as to perform control experiments under the same conditions with samples known to be non-superconducting to rule out experimental artifacts. Finally, it would also be useful to report the behavior of the complete hysteresis loops.

## Data Availability

No datasets were generated or analyzed during the current study.

## References

[1] Bud’ko, S.L., Huyan, S., Xu, M., Canfield, P.C.: Trapped flux in a small crystal of at ambient pressure and in a diamond anvil pressure cell. Supercond. Sci. Technol. **37**, 065010 (2024)

[2] Minkov, V.S., Ksenofontov, V., Budko, S.L., Talantsev, E.F., Eremets, M.I.: Magnetic flux trapping in hydrogen-rich high-temperature superconductors. Nat. Phys. **19**, 1293 (2023)

[3] Eremets, M.I.: The current status and future development of high-temperature conventional superconductivity. Nat. Sci. Rev. **11**, nwae047 (2024) 10.1093/nsr/nwae047 PMC11173201 38883300

[4] Hirsch, J.E., Marsiglio, F.: Evidence against superconductivity in flux trapping experiments on hydrides under high pressure. J. Supercond. Nov. Magn. **35**, 3141–3145 (2022)

[5] Hirsch, J.E., Marsiglio, F.: Further analysis of flux trapping experiments on hydrides under high pressure. Phys. C **620**, 1354500 (2024)

[6] Bud’ko, S.L., Xu, M., Canfield, P.C.: Trapped flux in pure and Mn-substituted and superconducting single crystals. Supercond. Sci. Technol. **36**, 115001 (2023)

[7] These fits were performed using the linear regression calculator at https://www.graphpad.com/quickcalcs/linear1/

[8] Kaluarachchi, Udhara S., Taufour, Valentin, Sapkota, Aashish, Borisov, Vladislav, Kong, Tai, Meier, William R., Kothapalli, Karunakar, Ueland, Benjamin G., Kreyssig, Andreas, Valentí, Roser, McQueeney, Robert J., Goldman, Alan I., Bud’ko, Sergey L., Canfield, Paul C.: “Pressure-induced half-collapsed-tetragonal phase in . Phys. Rev. B **96**, 140501 (2017)

[9] Hirsch, J.E., Marsiglio, F.: Nonstandard superconductivity or no superconductivity in hydrides under high pressure. Phys. Rev. B **103**, 134505 (2021)

